# Increasing macrolide resistance among *Streptococcus agalactiae* causing invasive disease in non-pregnant adults was driven by a single capsular-transformed lineage, Portugal, 2009 to 2015

**DOI:** 10.2807/1560-7917.ES.2018.23.21.1700473

**Published:** 2018-05-24

**Authors:** Elísia Lopes, Tânia Fernandes, Miguel P Machado, João André Carriço, José Melo-Cristino, Mário Ramirez, Elisabete R Martins, the Portuguese Group for the Study of Streptococcal Infections

**Affiliations:** 1 Institute of Microbiology, Institute of Molecular Medicine, Faculty of Medicine, University of Lisbon, Lisbon, Portugal; 2 These authors contributed equally to this work; 3 The members of group are listed at the end of the article

**Keywords:** antimicrobial resistance, bacterial infections, epidemiology, invasive streptococcal infections, molecular methods, Streptococcus agalactiae

## Abstract

We characterised Lancefield group B streptococcal (GBS) isolates causing invasive disease among non-pregnant adults in Portugal between 2009 and 2015. All isolates (n = 555) were serotyped, assigned to clonal complexes (CCs) by multilocus sequence typing and characterised by surface protein and pilus island gene profiling. Antimicrobial susceptibility was tested by disk diffusion and resistance genotypes identified by PCR. Overall, serotype Ia was most frequent in the population (31%), followed by serotypes Ib (24%) and V (18%). Serotype Ib increased significantly throughout the study period (p < 0.001) to become the most frequent serotype after 2013. More than 40% of isolates clustered in the CC1/*alp*3/PI-1+PI-2a genetic lineage, including most isolates of serotypes Ib (n = 110) and V (n = 65). Erythromycin and clindamycin resistance rates were 35% and 34%, respectively, both increasing from 2009 to 2015 (p < 0.010) and associated with CC1 and serotype Ib (p < 0.001). The Ib/CC1 lineage probably resulted from acquisition of the type Ib capsular operon in a single recombination event by a representative of the V/CC1 macrolide-resistant lineage. Expansion of the new serotype Ib/CC1 lineage resulted in increased macrolide resistance in GBS, causing invasive disease among adults in Portugal. The presence of this clone elsewhere may predict more widespread increase in resistance.

## Introduction

*Streptococcus agalactiae*, or Lancefield group B streptococci (GBS), are recognised as part of the human microbiota and a leading cause of neonatal infections [1]. In the past decades, it has grown in importance as a causative agent of invasive disease among non-pregnant adults worldwide [2-4]. Ageing of the human population and underlying medical conditions such as diabetes mellitus are likely to be contributing to the increasing number and severity of GBS infections in adults [2]. GBS disease may range from mild urinary tract infections to more severe conditions such as skin and soft tissue infections or potentially life-threatening syndromes such as bacteraemia without a focus, endocarditis and meningitis [2].

The first line antibiotic for prophylaxis and treatment of GBS disease is penicillin, to which GBS are considered universally susceptible. However, cases of reduced penicillin susceptibility associated with mutations in the penicillin-binding proteins (PBPs) have been described [5], raising concern about emergence of beta-lactam resistance, but the clinical significance of these findings is still poorly defined. Clindamycin is a suitable alternative for therapy of GBS infections in patients allergic to penicillin. However, in the past decade, significant increases in macrolide and lincosamide resistance rates in both neonatal and adult invasive infections were reported worldwide [3,4,6]. The emergence and dissemination of resistant clones may reflect the impact of long-term use of antibiotics as intrapartum prophylaxis to prevent GBS neonatal disease, as well as of the high doses and extended treatment courses for older individuals.

The capsular polysaccharide (CPS) is a major virulence factor and a target of the GBS vaccines currently under development [7]. To date, 10 capsular serotypes have been identified (Ia, Ib, and II–IX); their prevalence is known to vary both temporally and geographically. Sequence-based methods such as multilocus sequence typing (MLST) are now broadly used to further discriminate GBS isolates into genetic lineages that have been shown to differ in disease potential and tropism [8]. Nevertheless, GBS infections are mostly caused by a limited number of genetic lineages and serotypes. Particular serotype/genotype combinations were identified as leading causes of invasive GBS disease worldwide, namely the serotype III hypervirulent lineage among neonates, represented by clonal complex (CC) 17 [9]. Among adults, serotype V, mostly belonging to CC1 and expressing macrolide resistance, has been associated with invasive disease in Europe and the United States (US) [2,10] but more recently, other serotypes have gained relevance in this context. Serotype III was reported as the most frequent among invasive disease cases in adults in Norway [10], France [11] and Canada [12]. In contrast, in England and Wales [3] and in a previous study in Portugal [13], serotype Ia was dominant, demonstrating that there may be differences in the prevalence of serotypes between different countries. Despite the clonality observed within GBS populations causing invasive disease, increasing diversity within these homogenous genetic lineages reveals some extent of serotype exchange. While the driving forces behind capsular switching are not clear, a future introduction of CPS-based vaccines may exert further pressure driving the emergence of non-vaccine serotypes, highlighting the importance of continued surveillance.

We undertook this study to document potential changes in clonal composition and antimicrobial susceptibility of adult GBS invasive disease in Portugal.

## Methods

### Bacterial isolates and serotyping

From 2009 to 2015, microbiology laboratories in 24 Portuguese hospitals were asked to submit to a central laboratory isolates responsible for GBS invasive infection in non-pregnant adults (≥ 18 years-old). Whenever isolates of the same patient were available from more than one normally sterile sample, only the first isolate was included in this study.

While our network comprised most of the hospital microbiology laboratories in Portugal, this study was based on voluntary reporting and is therefore not population-based. Also, no audits to monitor compliance of the reporting laboratories were performed, so it is possible that not all cases of laboratory-confirmed invasive GBS disease occurring within our surveillance network were reported.

Capsular serotyping (Ia, Ib, II-IX) was done by the slide agglutination IMMULEX STREP-B Kit (Statens Serum Institute, Copenhagen, Denmark).

### Antimicrobial susceptibility testing and macrolide resistance phenotype

Susceptibility testing was performed by disc diffusion according to the Clinical and Laboratory Standards Institute (CLSI) methods and interpretation criteria [14]. All isolates were tested for susceptibility to: penicillin G, erythromycin, clindamycin, vancomycin, chloramphenicol, levofloxacin, gentamicin, streptomycin and tetracycline. A disk diffusion screening test for high-level aminoglycoside resistance (HLAR) was also performed according to the CLSI methods and interpretative criteria for *Enterococcus* species [14].

Macrolide resistance phenotypes were determined by a double-disk test according to CLSI guidelines [14]. Isolates were classified as expressing the M phenotype when they were resistant to macrolides only, or to the MLS_B_ phenotype when showing cross-resistance to macrolides and lincosamides, either constitutive (cMLS_B_) or inducible (iMLS_B_).

### Genotyping

Total bacterial DNA was isolated by treatment of the cells with mutanolysin and boiling. Among macrolide-resistant isolates, the presence of the *erm*(B), *erm*(A) (*erm*(TR) subclass), *erm*(T), and *mef* (*mef*(A) or *mef*(E)) genes was detected by multiplex PCR as previously described [15]. The presence of lincosamide resistance genes *lsa*(C) and *lnu*(B) was also tested by PCR [15].

Tetracycline-resistant isolates were screened for the presence of the *tet*(K), *tet*(L), *tet*(M), and *tet*(O) genes [15]. Among aminoglycoside resistant isolates, the presence of high-level resistance (HLR) determinants *aac*(*6’*)-*aph*(*2”*), *aph*(*2”*)-*Ib*, *aph*(*2”*)-*Ic*, *aph*(*2”*)-*Id*, *aph*(*3’*)-*III*, *ant*(*4’*)-*Ia* and *ant*(*6*)-*Ia* was tested by PCR [16,17].

MLST was performed as previously described [8] and allele numbers and sequence types (STs) were assigned by using the *S. agalactiae* MLST database (http://pubmlst.org/sagalactiae). Alleles and STs not previously described were deposited in the *S. agalactiae* MLST database. The goeBURST algorithm implemented in PHYLOViZ software [18] was used to establish relationships between STs. CCs were defined at the single-locus-variant (SLV) level.

The presence of GBS alpha (*bca*) and alpha-like (*eps*, *rib*, *alp*2, *alp3*, and *alp*4) surface protein genes [19,20] and of the pilus islands PI-1, PI-2a, and PI-2b [20] was evaluated by multiplex PCR.

### Typing analysis and statistics

Simpson’s index of diversity (SID) and 95% confidence intervals (CI) were calculated to evaluate diversity [21].

Differences and associations were evaluated by Fisher’s exact test with the false discovery rate (FDR) correction for multiple testing [22]. The Cochran–Armitage test (CA) was used for trends. A p value ≤ 0.05 was considered significant for all tests. Information about the Portuguese population in the study period (2009­–15) was obtained from Statistics Portugal [23].

### High-throughput sequencing

Two GBS isolates, representing ST1 serotype V (SH4916 isolate, SRR5575010) and Ib (SH5446 isolate, SRR5575009) were sequenced using the MiSeq system (Illumina, San Diego, US) and analysed by both mapping and de novo assembly approaches. The draft genomes and detailed methods can be found at http://dx.doi.org/10.6084/m9.figshare.5081992.

## Results

### Isolates

A collection of 555 GBS invasive isolates was recovered from patients with an average age of 69 years (range: 18–98 years). To analyse these data, cases were divided into two subpopulations: younger adults (18–64 years; n = 195; 35%) and older adults (≥ 65 years; n = 360; 65%). A similar number of isolates was recovered from men (n = 279; 50.3%) and women (n = 276; 49.7%). GBS were isolated from blood (n = 468), ascitic fluid (n = 35), synovial fluid (n = 29), cerebrospinal fluid (CSF) (n = 11), pleural effusion (n = 10), and aqueous humour (n = 2).

**Figure 1:**
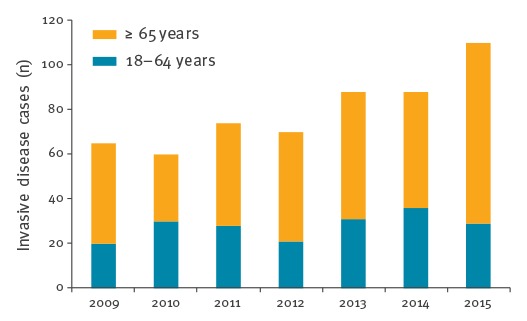
Number of group B streptococcal invasive infections, Portugal (24 hospitals), 2009–2015 (n = 555)

Overall, there was a higher frequency of GBS invasive disease among older than younger adults (overall incidence rate ratio (IRR) = 6.32; 95% CI: 5.32–7.73). During the study period, the number of infections among younger adults remained stable while GBS infections increased significantly among older adults (p (CA) < 0.001) (Figure 1).

### Serotype distribution

**Figure 2:**
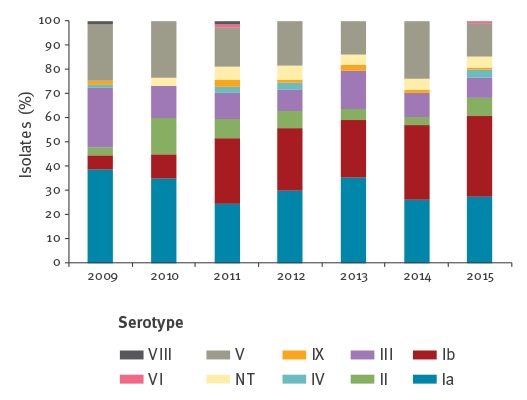
Serotype distribution of group B streptococcal isolates, Portugal (24 hospitals), 2009–2015 (n = 555)

Overall, there was significant serotype diversity (SID = 0.795; 95% CI: 0.780–0.810), with no significant difference in diversity between the two age groups. Serotype Ia was the most frequently found in the population (n = 169; 30.5%), followed by serotypes Ib (n = 133; 24.0%), V (n = 102; 18.4%), III (n = 70; 12.6%), II (n = 37; 6.7%), IV (n = 9; 1.6%), IX (n = 8; 1.4%), VI (n = 2; 0.4%) and VIII (n = 2; 0.4%). Serotype VII was not detected and 4.1% (n = 23) of the isolates were considered non-typeable. No associations were found between particular serotypes and age groups or sex. However, significant variations were observed in the relative proportion of serotypes throughout the study period (Figure 2). Serotype Ib increased significantly (p (CA) < 0.001), whereas serotype III decreased (p (CA) = 0.014). The decrease of serotype Ia from 38% in 2009 to 27% in 2015, was not statistically supported.

### Genetic lineages

The isolates were distributed across 57 STs (SID = 0.840; 95% CI: 0.815–0.865) and grouped into 10 CCs and two singletons. Twenty novel STs (ST768, ST769, ST772-ST779, ST781, ST810, ST815-ST818, ST875-ST877 and ST879) were identified in this study. The distribution of STs, serotypes, surface protein and pilus genes across clonal complexes is shown in Table 1.

**Table 1:** Distribution of sequence types, serotypes, surface protein and pilus genes of group B streptococcal isolates within clonal complexes, Portugal (24 hospitals), 2009–2015 (n = 555)

CC/ST (n)	Serotype (n)	Alp gene (n)	Pilus (n)
**CC1 (224)** ST1 (196), ST2 (13), ST196 (3), ST153 (2), ST499 (2), ST778 (1), ST810 (1), ST777 (1), ST815 (1), ST817 (1), ST818 (1), ST877 (1), ST879 (1)	Ib (110), V (77), Ia (12), IV (5), II (3), VI (2), NT (15)	*alp3* (202), *eps* (23), *bca* (5), *rib* (1)	PI-1+PI-2a (209), PI-2a (12), PI-1+PI-2b (5), PI-2b (2)
**CC4 (6)** ST3 (5), ST4 (1)	Ib (3), Ia (2), NT (1)	*bca* (5), *eps* (1)	PI-1+PI-2a (5), PI-2b (1)
**CC7 (1)** ST7 (1)	Ia (1)	*bca* (1)	PI-1+PI-2b (1)
**CC10 (37)** ST10 (18), ST8 (16), ST12 (3)	Ib (20), Ia (5), II (5), V (2), NT (5)	*bca* (35), *eps* (2)	PI-1+PI-2a (37)
**CC17 (32)** ST17 (22), ST109 (4), ST290 (2), ST291 (2), ST147 (1), ST779 (1)	III (31), IV (1)	*rib* (32)	PI-1+PI-2b (29), PI-2b (3)
**CC19 (73)** ST19 (36), ST28 (17), ST347 (9), ST110 (4), ST286 (2), ST27 (1), ST182 (1), ST267 (1), ST327 (1), ST335 (1), ST472 (1), ST529 (1), ST769 (1), ST772 (1), ST816 (1), ST876 (1)	III (37), II (27), V (10), Ia (1), IV (1), VIII (1), NT (2)	*rib* (73), *eps* (5), *bca* (1)	PI-1+PI-2a (74), PI-2a (4), PI-1+PI-2b (1)
**CC23 (157)** ST23 (79), ST24 (38), ST498 (18), ST144 (10), ST88 (3), ST640 (2), ST452 (1), ST756 (1), ST768 (1), ST774 (1), ST776 (1), ST781 (1), ST875 (1)	Ia (146), V (5), II (2), III (2), IV (2)	*eps* (81), *bca* (58), *rib* (11), *alp2* (7)	PI-2a (149), PI-1+PI-2a (7), PI-2b (1)
**CC26 (7)** ST26 (7)	V (7)	None (7)	PI-2a (7)
**CC103 (2)** ST103 (2)	Ia (2)	*bca* (2)	PI-2b (2)
**CC130 (8)** ST130 (8)	IX (8)	*bca* (8)	PI-2a (8)
**Singleton (2)** ST773 (1) ST775 (1)	V (1) VIII (1)	*eps* (1) *alp3* (1)	PI-1+PI-2a (1) PI-1+PI-2b (1)

Alp: alpha/alpha-like protein; CC: clonal complex; NT: non-typeable; ST: sequence type.

Despite the diversity of serotypes and STs, a small number of genetic lineages was responsible for most infections. More than 40% of the isolates represented CC1 (224/555), including most isolates of serotypes Ib (110/133; 82.7%) and V (65/102; 63.7%), all sharing surface protein gene *alp3* and the combination of PI-1 and PI-2a (Table 1). CC1 rose from accounting for 18% of the isolates in 2009 to 47% in 2015 (p (CA) < 0.001). The serotype Ib typically defined by CC10/*bca*/PI-1+PI-2a [8] represented only a small fraction of the isolates (n = 20) in this collection. A small number of serotype V isolates (n = 7) represented CC26/PI-2a, a genetic lineage lacking the *alp* surface protein gene. Serotypes II and III isolates represented mainly ST28 and ST19, respectively, of the CC19/*rib*/PI-1 + PI-2a genetic lineage. CC19 decreased in frequency in the study period (p (CA) = 0.006), in agreement with the observed decline among serotype III isolates. A smaller number of serotype III isolates was also part of the hypervirulent clone defined by CC17/*rib*/PI-1+PI-2b, frequently associated with neonatal invasive disease. Serotype Ia comprised mostly CC23, distributed into two sub-lineages defined by ST23/*eps*/PI-2a (n = 81) and ST24/*bca*/PI-2a (n = 51), and respective SLVs. CC23 accounted for 28% (157/555) of the isolates.

### Antimicrobial resistance

Antimicrobial resistance results are presented in Table 2. All 555 isolates were susceptible to penicillin, vancomycin and gentamicin. Non-susceptibility to chloramphenicol and levofloxacin was identified in 1.8% (n = 10) and 0.5% (n = 3) of the isolates, respectively. High-level resistance to streptomycin was detected in four isolates, all harbouring *aph(3’)-III* and *ant*(*6*)-*Ia*. Tetracycline resistance was found in 86.1% of the collection (n = 478), mostly associated with the *tet*(M) gene, but the *tet*(O) and *tet*(L) genes were also identified (Table 2).

**Table 2:** Distribution of antimicrobial resistance across group B streptococcal serotypes, Portugal (24 hospitals), 2009–2015 (n = 555^a^)

Serotype	Macrolides		Tetracycline	Streptomycin	Other (n)
	Phenotype (n)	Genotype (n)	Genotype (n)	Genotype (n)	
Ia	cMLS_B_ (9), iMLS_B_ (2), M (6)	*erm*B (7), *erm*TR (3), *erm*B+*lsa*C (1), *mef*E (6)	*tet*M (155), *tet*O (2), *tet*M+*tet*O (2)	*aph*(*3’*)-*III*+*ant (6)-Ia* (1)	CHL (1), LEV (2)
Ib	cMLS_B_ (110), iMLS_B_ (4)	*erm*B (105), *erm*TR (5), *erm*T (1), *erm*B+*erm*TR (1), *erm*B+*lsa*C (1), *erm*B+*mef*E (1)	*tet*M (122), *tet*M+*tet*O (4)	ND	CHL (4)
II	iMLS_B_ (2)	*erm*TR (1), *erm*T (1)	*tet*M (31)		None
III	cMLS_B_ (6), iMLS_B_ (10)	*erm*B (4), *erm*TR (11), *erm*B+*mef*E (1)	*tet*M (56), *tet*M+*tet*O (3), *tet*O (2)	*aph*(*3’*)-*III*+*ant (6)-Ia* (3)	CHL (3)
IV	None	ND	*tet*M (6), *tet*M+*tet*O (1)	ND	None
V	cMLS_B_ (25), iMLS_B_ (10)	*erm*B (24), *erm*TR (11)	*tet*M (67), *tet*M+*tet*O (4), *tet*M+*tet*L (1), *tet*O (2)	ND	CHL (1)
VI	None	ND	ND	ND	LEV (1)
VIII	None	ND	*tet*M (2)	ND	None
IX	M (1)	*mef*E (1)	ND	ND	CHL (1)
NT	cMLS_B_ (8), iMLS_B_ (2)	*erm*B (8), *erm*TR (2)	*tet*M (18)	ND	None

CHL: chloramphenicol; LEV: levofloxacin; M: resistance to macrolides; MLS_B_: resistance to macrolides, lincosamides and streptogramins B with the prefix letter referring to the constitutive (cMLS_B_) or inducible (iMLS_B_) expression of this phenotype; ND: not determined (resistance genotypes were not determined for the susceptible isolates); NT: non-typeable.

^a^ Only the isolates resistant to tested antimicrobial drugs are listed (n = 490).

**Figure 3:**
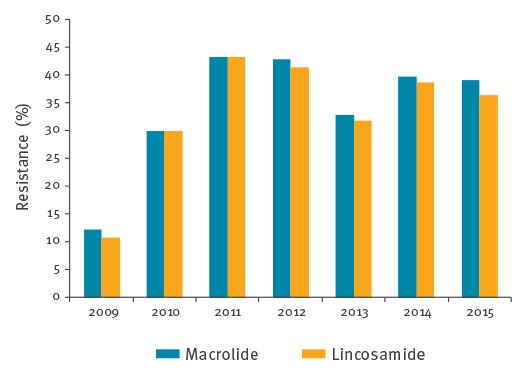
Macrolide and lincosamide resistance rates of group B streptococcal isolates, Portugal (24 hospitals), 2009–2015 (n = 555)

The overall rate of erythromycin resistance was 35.1% (n = 195) and of clindamycin 33.9% (n = 188). Both macrolide and lincosamide resistance increased throughout the study period (p (CA) = 0.005 and p (CA) = 0.010, respectively) (Figure 3). Most isolates were represented by the cMLS_B_ phenotype (n = 158), followed by iMLS_B_ (n = 30) and M (n = 7) phenotypes. While the *erm*(B) gene was exclusively found among the cMLS_B_ isolates, occasionally in association with other resistance determinants, the *erm*(TR) and *mef*(E) genes were the most frequent among iMLS_B_ and M phenotypes, respectively. Macrolide resistance was significantly associated with CC1 (p < 0.001), but only serotype Ib was overrepresented among resistant isolates (p < 0.001), with 98% (108/110) being macrolide-resistant, whereas among serotype V CC1 isolates only 35% (27/77) were resistant.

### Genomic analysis

**Figure 4:**
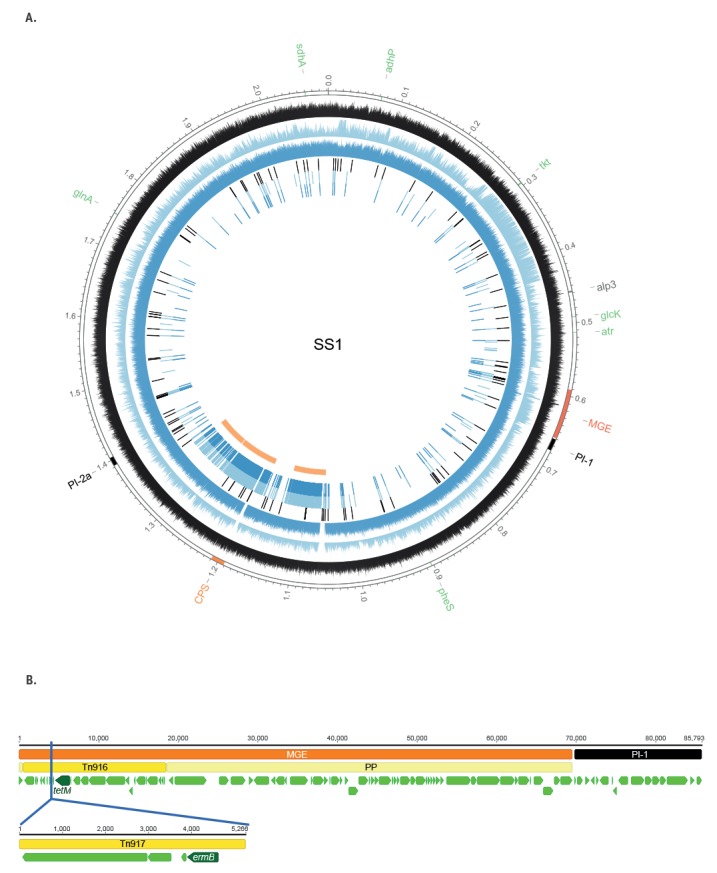
Genomic comparison of group B streptococcal isolates Ib/ST1 (SH5446) and V/ST1 (SH4916) to the SS1 genome

To characterise the putative capsule Ib switching in the ST1 background, we randomly chose one Ib/ST1 isolate (SH5446) and one V/ST1 isolate (SH4916) to examine at genomic level. The genomes were 2,057,595 bp and 2,065,479 bp long, respectively. Genomic analysis confirmed that both contained the genes encoding the Alp3 protein, PI-1 and PI-2a, and the corresponding capsular polysaccharide loci (Figure 4). We compared these two isolates from Portugal to the genomes of a serotype Ib/ST1 invasive isolate identified in Canada (NGBS217) [24] and the serotype V/ST1 strain SS1 [25]. The distribution of single-nucleotide polymorphisms (SNPs) and recombination regions is depicted in Figure 4. After removal of regions of recombination, SH5446 (Ib/ST1) and SH4916 (V/ST1) differed from each other by 94 SNPs, but presented 122 and 110 SNPs, respectively, relative to SS1 and differed by 55 and 105 SNPs, respectively, from the Canadian strain (NGBS217). In these three strains (SH5446, SH4916 and NGBS217), macrolide and tetracycline resistance co-localised in a mobile genetic element comprising a prophage, known to be present upstream of PI-1 [26], and the Tn3872 conjugative transposon. Tn3872 itself is composed of Tn917, carrying the *erm*(B) gene inserted into Tn916, which includes the *tet*(M) gene [27]. In contrast, strain SS1 carried only the Tn916 transposable element without the Tn917 insertion (Figure 4).

Alp: alpha/alpha-like protein; CDS: coding sequence; MGE: mobile genetic element; MLST: multilocus sequence typing; PP: prophage; SNP: single-nucleotide polymorphism.

Panel A. Whole genome SNP analysis. Outermost open circle (in grey) shows the SS1 genome. Several loci are represented: in green the MLST genes; in dark orange the capsular locus; in black the pilus loci; in red an MGE; in grey the locus encoding the Alp3 surface protein. The next three circles show the coverage depth of the SS1 genome and the other three circles display the SNP distribution. For all these six circles, the black circles represent ST1 serotype V isolate SH4916, the light blue the ST1 serotype Ib isolate NGBS217 and the dark blue the ST1 serotype Ib isolate SH5446. In the innermost circle, the recombination regions are displayed in orange.

Panel B. The MGE (next to PI-1 locus) into which the Tn917 transposable element carrying the *erm*(B) gene was inserted. The upper sequence represents the DNA fragment found in the SS1 genome. The PI-1 locus is represented in black and the MGE in red. The PP into which the Tn916 transposable element (in dark yellow) is inserted is represented in light yellow. CDS are represented by green arrows with the arrow tips showing the CDS orientation. The *tet*(M) gene found in Tn916 is represented in dark green. The blue vertical line represents the insertion position of the Tn917 transposable element in both ST1 serotype Ib samples (NGBS217 and SH5446), as well as in SH4916 (ST1 serotype V). The bottom sequence represents the inserted DNA fragment, the Tn917 transposable element (in dark yellow), found in the SH5446 isolate by de novo assembly. The *erm*(B) CDS is highlighted in dark green (the other large CDSs correspond to the transposase and resolvase found in Tn917).

## Discussion

We have identified a significant increase in the number of GBS infections in older adults in recent years in Portugal, similar to what has been reported elsewhere [3,4,28]. The age composition of the population did not change across the study period. While the present study was based on voluntary reporting, the surveillance system is composed of most hospitals in Portugal likely to diagnose GBS invasive disease and has been stable for many years. Therefore, we believe that the observed increase in GBS infections reflects a true trend, although this surveillance may underestimate the occurrence of GBS disease.

There was considerable serotype diversity, as expected from a broad spectrum of disease presentations, comparable to that found in previous years (2001 to 2008) [13]. Serotypes VIII and IX, recently identified for the first time in Portugal among neonatal invasive disease cases [15], were also represented in this collection, suggesting their recent introduction in the country.

There were significant changes in the prevalence of serotypes in the study period. Most striking was the increase of serotype Ib isolates, which accounted for only 8.9% from 2001 to 2008 [13] but became the most frequent serotype after 2013, reaching 34% of all infections in 2015. Capsular types Ia, III and V are responsible for the majority of GBS invasive disease cases in most countries [2,3,11,12], even though they may rank differently in time and geographic location. Serotype Ib has not been among the most frequent serotypes associated with invasive disease in adults and has been reported as accounting for 9–12% of isolates in the US, Canada, France, England and Wales [2,3,11,12]. In Portugal, its prevalence among neonatal infections has been equally unremarkable, accounting for 5.5% of cases [15].

A parallel rise in CC1, including most serotype Ib isolates, accompanied the increase of serotype Ib. For more than a decade, since the MLST scheme was first published [8], CC1 has mostly been associated with serotype V whereas CC10, grouping ST8, ST10 and ST12, is the CC most frequently found among serotype Ib isolates, regardless of disease type, age group or geographic origin [8,28,29]. Therefore, the grouping within CC1 of most isolates presenting serotypes Ib and V was unusual. Together with the fact that both these serotypes were represented by the alpha-like surface protein gene *alp3* and PI-1 and PI-2a, previously almost exclusively found associated with serotype V, this suggested that a capsular switching event could have created this new genetic lineage. In fact, in a recent study focusing on GBS neonatal infections in Portugal, we had already detected seven serotype Ib isolates within CC1, hypothesising their recent origin through capsular switching [15]. A recent report from Japan found serotype Ib as the most frequent among invasive disease cases in nonpregnant adults. Most of them represented the characteristic CC10, but the authors also reported a small number of serotype Ib isolates representing CC1 [30]. Furthermore, two serotype Ib/ST1 strains have been identified in Canada, and genomic analysis supported their origin from horizontal transfer of the capsular type Ib locus into a serotype V strain [24,31].

Capsular switching is known to occur in GBS [20,32], and these events are illustrated by the presence of a small number of new serotype/genotype combinations in any given CC in which a particular serotype is dominant. To clarify the origin of Ib/CC1/*alp*3/PI-1+PI-2a, we performed a genomic analysis of two representative isolates. We found a very small number of SNPs in our serotype Ib/ST1 isolate and the Canadian NGBS217 strain relative to the serotype V/ST1 [24] and Tn3872 that has been frequently detected among CC1 GBS isolates [25,33] was present in all three strains, which confirms that they shared the same genetic background of the widely disseminated serotype V clone. Recombination analysis performed with the Gubbins software indicated that three adjacent recombination events may have taken place to generate the new serotype Ib/ST1 strains. However, similar to what was suggested for the NGBS217 strain [24], the most parsimonious explanation is that a single recombination event was responsible for the replacement of a ca 300 kb DNA fragment including the type Ib capsular locus. Taken together, these studies support our hypothesis for the origin of this new genetic lineage and indicate that it is found in multiple countries. To date only represented by a few isolates outside Portugal, they may become established and expand in the future.

Given the wide geographic dissemination of the Ib/CC1 lineage, it is hard to say where it may have emerged. The small number of SNPs between the genomes of the Ib/ST1 isolates from Portugal and Canada and the larger number of SNPs relative to the genomes of serotype V isolates from the same locations, suggests a common origin of the Ib/ST1 isolates. These isolates are contemporary with those found since 2010 in neonatal invasive disease [15], indicating that the Ib/CC1 lineage causes infections in all age groups. Additional studies including more isolates may reveal further insights into the potential advantageous traits that the acquisition of such a large DNA fragment, including a novel capsular locus, may have introduced.

Within CC23, ST23 is frequently responsible for invasive disease cases in both neonates and adults, while ST24 is seldom found in most countries [28,29,34]. We have previously proposed that ST24 represents a successful clone within the geographical boundaries of the Mediterranean region given its high prevalence in Portugal and Spain [13,35]. Among neonatal invasive disease cases in Portugal, we found a higher frequency of ST24 within CC23, significantly associated with late-onset disease [15]. In this study, we also found a significant proportion of ST24 and its SLVs (ST498 and ST781) within serotype Ia/CC23 isolates (52/133; 39%); this is in agreement with this sublineage being well adapted and geographically mainly confined to the Mediterranean region.

Resistance rates to the antimicrobial drugs tested differed among serotypes and were unevenly distributed within the CCs. Erythromycin resistance (35.1%) increased throughout the study period (p (CA) = 0.005) and was significantly higher than that documented between 2001 and 2008 (12.9%) [13]. We hypothesise that an increase in macrolide use could explain the observed increase in resistance. In fact, macrolide consumption in the community increased in Portugal from 3.24 DHD in 1998 to 3.80 DHD in 2009, according to the Antimicrobial consumption database (ESAC-Net), [36]. However, such increases in consumption were accompanied by decreasing resistance among the related streptococcal species *S. pyogenes* and this trend was also noted in other geographic regions [37]. In contrast, since 2009, macrolide consumption in the community has decreased in Portugal and reached 3.02 DHD in 2015 [36], suggesting that antimicrobial consumption is only one of the selective pressures acting on antibiotic-resistant streptococci. Where increasing macrolide resistance in GBS was documented, this was associated with a high prevalence of serotype V [3,6,11]. However, in our study, resistance was associated with serotype Ib, indicating that the expansion of the Ib/CC1 lineage was the major driver in increasing macrolide resistance.

Serotype V was mostly associated with CC1, as was found elsewhere in infections in adults [3,12] although a small number of serotype V isolates (n = 7) represented CC26/PI-2a that appears to be common in African countries [38] and is infrequent in Europe. While the prevalence of serotype V changed with time, it did not show a significant trend in the study period. In contrast, the serotype Ib/CC1 genetic lineage emerged and expanded substantially, indicating that the new serotype/genotype combination is not replacing serotype V. It was suggested that the co-localisation of resistance and virulence genes within Tn3872 is consistent with the expansion of serotype V/ST1 GBS since the 1990s being facilitated by the acquisition of genetic determinants that contributed to an increased capacity to cause disease in non-pregnant adults [25]. It is unclear at present why the serotype Ib/CC1 macrolide-resistant lineage is expanding in Portugal, while it appears to be widely disseminated but not particularly prevalent elsewhere. Two major hypotheses could explain the success of this lineage: either the lineage has some fitness advantage achieved by specific combinations of genes that were transferred together with the capsular operon, increasing its ability to cause disease or persist through asymptomatic colonisation; or the selective pressures currently acting on GBS are particularly favourable to this clone. Further studies will be needed to clarify these points. Our results highlight the importance of the expansion of a single invasive GBS clone on the increase of antimicrobial resistance.

## Notes

**Members of the Portuguese Group for the Study of Streptococcal Infections:** Henrique Oliveira (Centro Hospitalar de Coimbra); Teresa Vaz, Marília Gião, Rui Ferreira (Centro Hospitalar do Barlavento Algarvio); Ana Cristina Silva, Hermínia Costa, Maria Fátima Silva, Maria Amélia Afonso (Centro Hospitalar de Entre Douro e Vouga); Ana Domingos, Gina Marrão, José Grossinho (Hospital de Santo André, Leiria); Paulo Lopes, Angelina Lameirão, Gabriela Abreu; Aurélia Selaru (Centro Hospitalar de Vila Nova de Gaia/Espinho); Hermínia Marques, Margarida Tomáz, Paula Mota (Centro Hospitalar do Alto Ave); Ezequiel Moreira, (Centro Hospitalar do Médio Ave); Maria Helena Ramos, Ana Paula Castro (Centro Hospitalar do Porto); Nuno Canhoto, Teresa Afonso (Hospital Central do Funchal); Teresa Pina, Helena Peres, Odete Chantre, João Marques, Cristina Marcelo, Isabel Peres, Isabel Lourenço, Margarida Pinto (Centro Hospitalar de Lisboa Central); Lurdes Monteiro, Luís Marques Lito (Centro Hospitalar Lisboa Norte); Cristina Toscano, Maria Ana Pessanha (Centro Hospitalar Lisboa Ocidental); Paulo Paixão, Cátia Piedade (Hospital dos Lusíadas, Lisboa); Elmano Ramalheira, Raquel Diaz, Sónia Ferreira, Inês Cravo Roxo (Centro Hospitalar do Baixo Vouga); Ana Paula Castro (Hospital de Vila Real); Graça Ribeiro, Rui Tomé, Celeste Pontes, Luísa Boaventura, Catarina Chaves, Teresa Reis (Hospitais da Universidade de Coimbra); Ana Buschy Fonseca (Hospital de Cascais); Manuela Ribeiro, Helena Gonçalves (Hospital de São João, Porto); Alberta Faustino, Adelaide Alves, Maria Cármen Iglesias (Hospital de Braga); Maria Luísa Gonçalves, Olga Neto (Hospital dos SAMS, Lisboa); Luísa Sancho (Hospital Dr. Fernando da Fonseca, Amadora/Sintra); Adriana Coutinho (Hospital do Espírito Santo, Évora); José Diogo, Ana Rodrigues (Hospital Garcia de Orta, Almada); Maria Antónia Read, Valquíria Alves, Margarida Monteiro (Hospital Pedro Hispano, Matosinhos); Rosa Bento (Unidade Local de Saúde do Baixo Alentejo, Beja); Vitória Rodrigues, Patrícia Pereira, Sofia Marques, Joana Selada (GeneralLab, Lisboa); Paulo Paixão (Hospital da Luz, Lisboa); Elsa Calado (Unianálises, Porto).
